# Mirizzi Syndrome With Bouveret Syndrome: A Rare Amalgam

**DOI:** 10.7759/cureus.24187

**Published:** 2022-04-16

**Authors:** Vaibhav K Varshney, Sabir Hussain, B. Selvakumar, N. Vignesh, Binit Sureka

**Affiliations:** 1 Surgical Gastroenterology, All India Institute of Medical Sciences, Jodhpur, IND; 2 Gastroenterology, Dr. Sampurnanand Medical College, Jodhpur, IND; 3 Radiology and Interventional Radiology, All India Institute of Medical Sciences, Jodhpur, IND

**Keywords:** open surgical gastrostomy, duodenal fistula, bouveret's syndrome, surgical obstructive jaundice, chronic cholecystitis, chronic cholelithiasis

## Abstract

Mirizzi and Bouveret syndromes are uncommon but important complications of calculous cholecystitis. Mirizzi syndrome commonly presents with jaundice due to extrinsic compression on the common bile duct by an impacted stone at the gall bladder infundibulum, whereas Bouveret syndrome presents with gastric outlet obstruction due to a large stone in the duodenum. Our case is a 65-year-old lady who presented with pain in the right upper abdomen associated with nausea and vomiting. Contrast-enhanced computed tomography and MRI of the abdomen were suggestive of calculus in the infundibulum of the gall bladder with compression over the common bile duct and a large stone in the first part of the duodenum. Upper gastrointestinal endoscopy confirmed the findings but could not retrieve the stone. Cholecystectomy with the retrieval of calculus from the infundibulum and duodenum was performed with the closure of the fistulous opening. The patient did well in the post-operative period and is doing well after nine months of follow-up. Chronic calculus cholecystitis can present in varied forms, and one should be aware of such rare complications and their management.

## Introduction

Cholelithiasis is a common disorder and may pose further problems when it causes luminal obstruction of either the bile duct or intestine. Mirizzi syndrome is an uncommon complication of cholelithiasis, and presentation varies from mild extrinsic compression on the common bile duct (CBD) to its fistulization leading to jaundice [1]. Bouveret syndrome, an unusual subset of gallstone ileus, is characterized by the passage of a stone from the gall bladder (GB) to the pylorus or duodenum through an acquired bilioenteric fistula and causes gastric outlet obstruction (GOO) [2]. We hereby present a unique case with a combination of both these syndromes in an elderly lady who was managed successfully.

## Case presentation

A 65-year-old lady presented with a complaint of pain in the right upper abdomen, which was mild in intensity and gradually progressive over the past 15 days. She had nausea with intermittent episodes of non-bilious vomiting; however, she could tolerate a liquid diet. There was no history of fever, jaundice, anorexia, weight loss, awareness of lump, decreased urine output, or altered bowel habits. She also had no similar complaints in the past. General physical examination showed no pallor, icterus, or lymphadenopathy. Her abdominal examination was unremarkable except for a succussion splash in the upper abdomen. Her hemogram, liver function test, and electrolytes were within normal limits.

**Figure 1:**
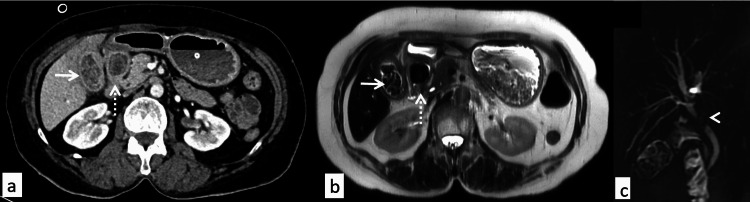
Cross-sectional images (A) Axial contrast-enhanced CT images showing multiple gallstones in the thickened gallbladder (arrow) with thickening of adjacent duodenum containing hyperdense contents (dashed arrow). (B) Axial T2-weighted MRI confirming the presence of gallstones (arrow) and large hypointense stone within the duodenal lumen (dashed arrow). (C) Magnetic resonance cholangiopancreatography image showing mild biliary dilatation with signal loss (arrowhead) due to extrinsic compression in proximal common bile duct resulting in Mirizzi syndrome.

She was evaluated with an ultrasound of the abdomen, which was suggestive of a distended GB and thickened wall with multiple calculi noted within. Contrast-enhanced computed tomography (CECT) of the abdomen reported thickened GB wall with a linear tract extending from the neck of the GB into the first part of the duodenum (D1). A large calculus of approximately 1.9 x 1.8 cm was noted in the D1, causing dilatation of the stomach. Another calculus of approximately 10 x 7.0 mm was seen impacted in the neck region with associated mild intrahepatic biliary radical dilatation (Figures 1A, 1B). Magnetic resonance cholangiopancreatography (MRCP) was suggestive of cholelithiasis with diffuse irregular altered signal intensity in the GB wall, which was contiguous with the soft tissue lesion involving pyloroduodenal region and compression effect over CBD (Figure 1C).

**Figure 2:**
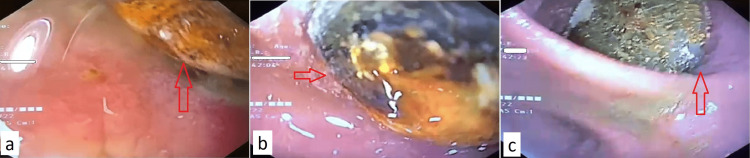
Upper esophagogastroduodenoscopy depicting impacted stone in the duodenum (arrow)

An upper gastrointestinal endoscopy was performed, which revealed dilated stomach with a large calculus seen just distal to the pylorus in D1. However, the calculus could not be retrieved using an extraction balloon and Dormia basket (Figures 2A-2C).

**Figure 3:**
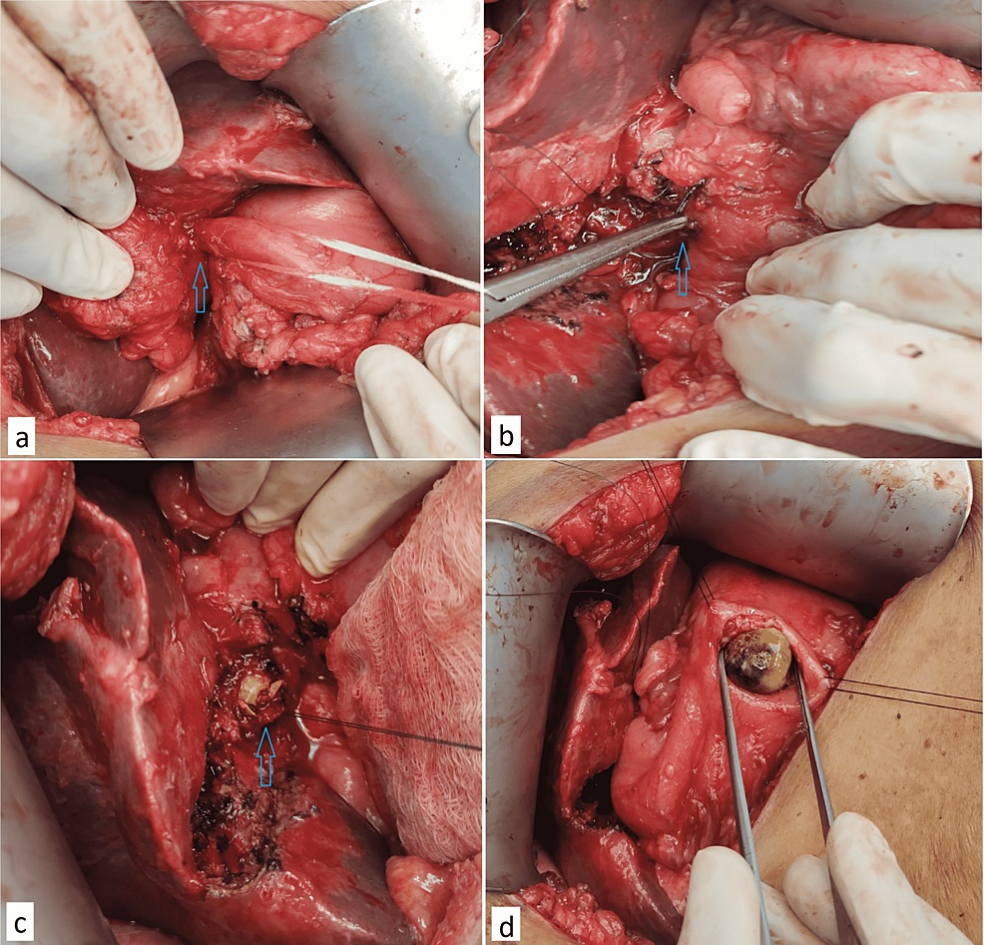
Intra-operative images (A-B) Cholecystoduodenal fistula looped with the help of umbilical tape (arrow) and right-angled artery forceps inside the fistulous opening of the duodenum (arrow). (C) Impacted calculus in the infundibulum of the GB. (D) Calculus being retrieved from the stomach.

The patient underwent laparotomy via right subcostal incision and was found to have omentum and transverse colon adherent to the GB and hepatoduodenal ligament. After releasing the adhesions, the GB was found distended and having multiple calculi with an impacted calculus in its infundibulum adherent to the CBD. Apart from this, the GB was also adherent to the first part of the duodenum with the formation of a cholecystoduodenal fistula (Figures 3A, 3B). Subtotal cholecystectomy was done by fundus-first technique with the dismantling of the fistula; the impacted infundibulum stone was removed, and the stump was closed after ensuring complete clearance. Further, an impacted stone (~2 cm) was noted in the first part of the duodenum, which was pushed gently into the stomach. A small gastrotomy was created, and the stone was retrieved (Figures 3C, 3D). After this, both gastrotomy and the small duodenal fistulous opening were closed in two layers using interrupted polydioxanone sutures. Hence, the diagnosis of Mirizzi syndrome (type Vb) with Bouveret syndrome was made.

The patient had a high nasogastric tube output of ~500 ml/day on the first post-operative day (POD), which gradually decreased over three days. She was allowed oral intake on the fourth POD, which she tolerated well. She had a minimal bile leak of ~100 ml/day from the drain on the second POD, which gradually decreased on conservative management, and the drain was removed on the seventh POD. She was discharged on the eighth POD on a full oral diet and is doing well after nine months of follow-up.

## Discussion

Mirizzi syndrome is an unusual complication of cholelithiasis encountered in 0.05-5.7% of patients [3]. It develops due to the impacted calculus in the infundibulum of GB, leading to extrinsic compression of the CBD with associated chronic inflammation; it most commonly presents with jaundice and cholangitis [1,3]. Over time, it may erode into the CBD, forming a cholecystobiliary fistula of various levels. As per the classification proposed by Csendes in 1989 and validated by Beltran et al. in 2008, any variant of Mirizzi syndrome in association with a cholecystoenteric fistula is classified as type V [4]. It is further sub-classified as type Va and Vb based on the absence or presence of associated gallstone ileus, respectively.

Independently, in long-standing cholelithiasis, the calculi may enter the gut through a cholecystoenteric fistulous tract between the GB and stomach, duodenum, or colon and labeled as gallstone ileus, which happens in nearly 0.3-0.5% of all cases of cholelithiasis [5,6]. Usually, the site of obstruction in gallstone ileus is the terminal ileum; but rarely, it can happen at the pylorus or duodenum in 1-4% of cases [5]. This form of proximal gallstone ileus is termed Bouveret syndrome. In the present case, we encountered a rare combination of both Mirizzi syndrome (Vb) and Bouveret syndrome, which has been reported once in the literature [7]. Chiang and Ryou reported a case with the presence of both cholecystoduodenal fistula and communication between the GB and the common hepatic duct; however, surgical details and images were not shared, making our case distinct from it [7].

The management of Mirizzi syndrome usually begins with biliary drainage with or without stone retrieval, as the most common presentation is obstructive jaundice. ERCP with stent placement allows decompression of the CBD in patients with jaundice or cholangitis. Though less frequent, endoscopic retrieval of CBD calculi may eliminate the need for surgical CBD exploration. Open surgery is the current standard for Mirizzi syndrome. Laparoscopic surgery may be attempted in experienced centers but has a high conversion rate of 11-80% [1]. Usually, for type I and II Mirizzi syndrome, subtotal cholecystectomy is preferred as there is significant scarring in the Calot’s triangle, and the remnant GB is closed as a choledochoplasty with absorbable suture. A bilioenteric anastomosis, preferably a Roux-en-Y hepaticojejunostomy, is required for type III and IV Mirizzi syndrome as more than one-third of the wall is already destroyed by the disease process. However, its type V variant is treated with the division of the bilioenteric fistula, subtotal cholecystectomy, and closure of the fistula, as was performed in the present case.

The prevalence of cholelithiasis and its associated complications increases with age; hence, Bouveret syndrome is usually encountered in elderly ladies over the age of 70 years [8]. The usual presentation is nausea, vomiting, abdominal pain, and rarely upper gastrointestinal bleeding. Patients may have features of dehydration such as tachycardia, low urine output, and metabolic alkalosis in acute settings. CECT and MRCP are the main diagnostic imaging modalities as they provide exact anatomical delineation of the fistulous tract, location of calculus, and level of obstruction. Rigler’s triad, which is composed of the presence of a gallstone in the duodenum, dilated stomach, and pneumobilia, is characteristic of Bouveret syndrome; however, all features are encountered only in one-fifth of cases [8]. In our case also, pneumobilia was not seen despite having cholecystoduodenal fistula.

The initial management is endoscopic therapy, which has gained popularity for Bouveret syndrome. The impacted calculus can be removed endoscopically with the help of a Dormia basket, mechanical or electrohydraulic lithotripsy, and extracorporeal shock wave lithotripsy for bigger stones [5]. Although less morbid, endoscopic stone extraction is successful in only 29% of patients [9]. Further, few patients have developed small bowel or colonic obstruction due to a stone fragment that has passed distally after endoscopic manipulation and subsequently required surgical intervention [10]. Hence, surgery remains the only option if endoscopic therapy fails or does not allow complete clearance of all calculi. The procedure involves cholecystectomy, enterolithotomy, dismantling, and closure of the concurrent fistula. The impacted gallstones can be removed through a duodenotomy or gastrotomy; the later route is preferred as stones are usually large and closure of the stomach is easy. As these patients are elderly with multiple comorbidities, if the patient is unstable, a two-stage approach is advocated to decrease the surgical morbidity and mortality: retrieval of stone in the duodenum to relieve the obstruction as the first emergency surgery followed by cholecystectomy with dismantling of the fistula as the second elective surgery; however, there is a 5% risk of recurrent gallstone ileus before the second surgery [11]. A laparoscopic approach is successful in removing stones up to 5 cm; however, this approach is usually difficult due to dense adhesions in the hepatoduodenal ligament.

## Conclusions

Both Mirizzi and Bouveret syndromes are rare complications of cholelithiasis and usually occur in elderly female patients. Mirizzi syndrome occurring together with Bouveret syndrome has been reported rarely. Accurate pre-operative diagnosis is by imaging and helps in surgical planning and avoiding complications. Open surgery is the current standard and involves subtotal cholecystectomy, dismantling of the fistula, and retrieval of the stone with additional biliary drainage if needed.
